# Exogenous Nitric Oxide Alleviates the Damage Caused by Tomato Yellow Leaf Curl Virus in Tomato through Regulation of Peptidase Inhibitor Genes

**DOI:** 10.3390/ijms232012542

**Published:** 2022-10-19

**Authors:** Xian Wang, Baoqiang Wang, Xiaolin Zhu, Ying Zhao, Baoxia Jin, Xiaohong Wei

**Affiliations:** 1 College of Agronomy, Gansu Agricultural University, Lanzhou 730070, China; 2 Gansu Provincial Key Laboratory of Aridland Crop Science, Lanzhou 730070, China; 3 Gansu Key Lab of Crop Genetic & Germplasm Enhancement, Lanzhou 730070, China; 4 College of Life Science and Technology, Gansu Agricultural University, Lanzhou 730070, China

**Keywords:** nitric oxide, tomato, transcriptome, TYLCV, resistance induction

## Abstract

The tomato yellow leaf curl virus (TYLCV) is the causal agent of one of the most severe diseases affecting tomato growth; however, nitric oxide (NO) can mediate plant resistance. This study investigated the molecular mechanism of exogenous NO donor-mediated disease resistance in tomato seedlings. Tomato seedlings were treated with sodium nitroprusside and TYLCV and subjected to phenotypic, transcriptomic, and physiological analyses. The results show that exogenous NO significantly reduced disease index, MDA content, and virus content (71.4%), significantly increased stem length and fresh weight of diseased plants (*p* < 0.05), and improved photosynthesis with an induction effect of up to 44.0%. In this study, it was found that the reduction in virus content caused by the increased expression of peptidase inhibitor genes was the main reason for the increased resistance in tomatoes. The peptidase inhibitor inhibited protease activity and restrained virus synthesis, while the significant reduction in virus content inevitably caused a partial weakening or shutdown of the disease response process in the diseased plant. In addition, exogenous NO also induces superoxide dismutase, peroxidase activity, fatty acid elongation, resistance protein, lignin, and monoterpene synthesis to improve resistance. In summary, exogenous NO enhances resistance in tomatoes mainly by regulating peptidase inhibitor genes.

## 1. Introduction

The tomato (*Solanum Lycopersicum*) is one of the fruit and vegetable crops gradually being grown around the world and is loved by the public for its unique taste, rich nutrition, and variety of consumption options [1,2]. In recent years, the tomato yellow leaf curl virus (TYLCV) has posed a significant threat to tomato cultivation. The disease is caused by the tomato yellow leaf curl virus (TYLCV), which is the most damaging of the top 10 plant viruses [3] and is mainly transmitted by whiteflies, so it is also known as Whitefly-transmitted geminivirus (WTG). Grafting can of course also lead to the spread of the virus [4,5]. TYLCV contains a single-stranded circular DNA molecule of 2.6 to 2.8 kb and is classified as a *begomovirus*. This virus encodes six open reading frames (ORFs): *C1*, *C2*, *C3*, *C4*, *V1,* and *V2*. These genes are involved in the encoding or regulation of replication-associated protein (Rep), transcriptional activator protein (TrAP), replication enhancer protein (REn), pathogenicity, coat protein (CP), viral movement and suppression of host immunity, respectively [6,7,8,9,10]. Once infected with this virus, tomatoes will suffer from dwarfism, shortened stem node spacing, reduced biomass, yellowing and curling of the leaves, and fruit deformities, resulting in severe cases of a 100% reduction in yield and loss of commercial value [11]. The main strategies to combat the disease are breeding resistant varieties, the use of insecticides, chemicals, and field management [12]. However, these measures also have limitations such as high costs, pesticide residue problems, and low efficiency [13].

Nitric oxide (NO) is a multifunctional signaling molecule commonly found in plants, which can modify proteins, regulate the expression of pathogenesis-related protein 1 (PR1), and trigger the plant’s disease resistance defense system, thus helping the plant overcome adversity and ultimately achieve the purpose of virus resistance [14,15]. What is more important is that NO is inexpensive. Different concentrations of SNP (sodium nitroprusside, an NO donor) solutions have significant inhibitory effects on plant diseases, e.g., a concentration of 50 μmol/L can stimulate the synthesis of phenolic compounds, antioxidant enzyme activity, and defense enzyme activity by regulating the level of H_2_O_2_, thus enhancing the resistance of citrus fruits to *Colletotrichum gloeosporioides* [16]. Lu et al. [17] showed that SNP greatly reduced the incidence of the rice black-streaked dwarf virus (RBSDV) in rice plants by regulating gene expression, salicylic acid content, and protein S-nitrosylation. The improvement of tomato resistance to gray mold by an SNP solution at 0.02 mmol/L is associated with jasmonic acid-like substances and PAL enzyme activity [18]. SNP at 50, 100, and 200 μmol/L induced photosynthetic pigments, enzymatic activity, and thus improved peanut resistance to the peanut mottle virus (PeMV) [19].

In summary, exogenous NO enhances plant defense against pathogenic bacteria by regulating defense enzyme activities (peroxidase, POD; phenylalanine ammonia-lyase, PAL; polyphenol oxidase, PPO), antioxidant enzyme activities (ascorbate peroxidase, APX; catalase, CAT; superoxide dismutase, SOD), secondary metabolite content, and photosynthesis. It has also been shown that NO itself combines with O_2_.^−^ to produce peroxynitrite ions (ONOO^−^), a process that not only reduces the content of superoxide anions but also produces ONOO^−^ that is lethal to pathogenic microorganisms, thereby inhibiting them [20,21]. In addition, peptidase inhibitors also play an important role in plant disease resistance. For example, in the preliminary localization of tomato degreening virus disease resistance genes, Gao Wenzheng et al. [22] found that its expression was higher in disease-resistant materials than in sensitive materials.

RNA-seq sequencing technology is often used to study the molecular response mechanism of the plant–pathogen interaction process [23], and it is the best method to screen for disease-resistance genes as well as to find key disease-resistance processes and pathways. Chen et al. [24] showed that the defense response to the TYLCV in tomatoes was mainly manifested in cell wall reorganization, transcriptional regulation, defense response, ubiquitination, and metabolite synthesis, and that the gene expression levels of WRKY transcription factors, R genes, protein kinases, and receptor (-like) kinases were down-regulated in susceptible varieties. The results of the transcriptomic analysis showed that the expression of genes related to oxidative stress response was increased after tomato susceptibility and hormonal signaling pathways, such as ethylene, were regulated, which in turn induced defense responses [25], but studies on the physiological and molecular levels of the exogenous NO-induced inoculation of tomatoes with the TYLCV are scarce. Therefore, in this study, we selected the susceptible to TYLCV cultivar Jinpeng 1, exogenously sprayed SNP to induce disease resistance in the tomato, and performed a morphological observation, a physiological response, and a statistical analysis of gene expression at the transcriptional level to reveal the genes and pathways related to the process of NO-induced disease resistance in the tomato, to understand the potential mechanism of NO regulation of tomato disease resistance, and to provide new ideas for molecular breeding to develop disease-resistant varieties.

## 2. Results 

### 2.1. Virus Detection

**Figure 1:**
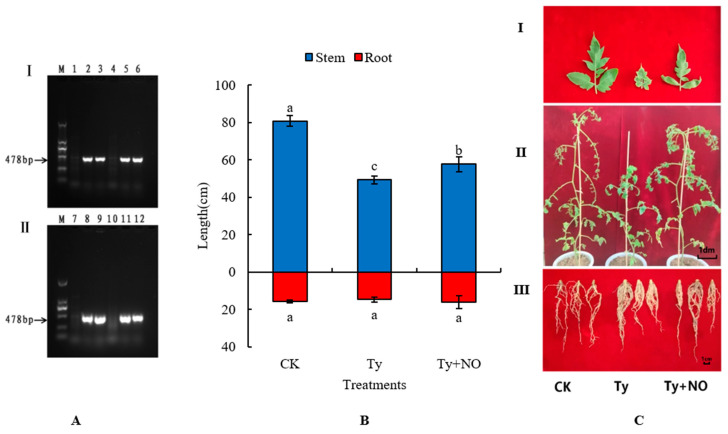
Changes in tomato inoculation and performance 30 d after inoculation. (**A**) TYLCV assay results at 9 d and 36 d after TYLCV inoculation. I and II indicate virus detection at day 9 and day 36 after inoculation, respectively. In 1–6 is indicated the detection results on day nine after virus inoculation, where 1 and 4 indicate the control group and 2, 3, 5, and 6 indicate the virus-inoculated group. In 7–12 is indicated the detection results on day 36 after virus inoculation, where 7 and 10 indicate the control group and 8, 9, 11, and 12 indicate the virus-inoculated group. (**B**) Effect of exogenous NO on plant height and root length at 30 d after tomato susceptibility. Different lowercase letters in the graph indicate significant differences (*p* < 0.05), the same below. (**C**) Effect of exogenous NO on phenotype at 30 d after tomato susceptibility. I, II and III indicate the effects of exogenous NO on leaves, stems and roots of virus-infected tomatoes, respectively.

Figure 1 shows the results of the virus detection under each treatment. It can be seen that the control group did not show a specific band, suggesting that the virus was not infected throughout the trial. In contrast, the virus treatment groups all successfully amplified a specific band (478 bp), indicating successful virus inoculation. Therefore, the conditions for the subsequent experiment were satisfied. 

### 2.2. Effect of Exogenous NO on Phenotypic and Growth Indicators of Virus-Inoculated Tomatoes

In the tomato–pathogen interaction, exogenous NO had a significant positive effect on the phenotype, with reduced symptoms after treatment with exogenous NO compared to the control. After 30 d post-inoculation, the plants inoculated by the TYLCV were severely dwarfed, the leaves were severely yellowed, crinkled and smaller, the stem length and root length were shortened, both dry and fresh weight were reduced, and stem diameter and root diameter increased, indicating the TYLCV had a significant effect on stem length, fresh weight, stem diameter, and root diameter (*p* < 0.05). However, in treatment with exogenous NO, a series of phenotypic symptoms caused by the TYLCV in tomato leaves were reduced, and stem length, dry weight, and fresh weight exhibited recovery (Figure 1B,C, Table 1), with significant recovery in stem length and fresh weight, which were 26.48% and 131.2%, respectively. In addition, root hairs were significantly increased in the positive control group, while tomato roots were significantly restored in the treated group (Figure 1C-III). This indicates that tomatoes showed a tendency of lateral growth after disease susceptibility in which exogenous NO broke.

### 2.3. Effect of Exogenous NO on the Control of TYLCV

As shown in Table 2, there were 20 tomato plants for each treatment, and the infection rate of the positive control group was 100%, while the infection rate of the control group was 0%. The severity of the disease was 62.5% at 30 d after Jinpeng 1 virus inoculation, while it was reduced to 35.0% after exogenous NO prophylaxis; the relative mimicry was as high as 44%. 

### 2.4. Transcriptome Sequencing Data and Analysis

To further understand the molecular mechanisms underlying the alleviation of TYLCV damage in tomatoes by exogenous NO, RNA-seq analysis was performed on a 20 d treatment inoculated with the virus after a comprehensive analysis of phenotypic and growth changes. The RNA quality detection was qualified, with all samples having concentrations greater than 462 ng/ul and all totaling greater than 17.56 μg (Table 3). In this study, the data of all nine samples reached more than 6 G. The raw data obtained from sequencing were filtered to obtain clean data, and the percentage of clean data was higher than 99.81% (Table S2). The base mass analysis showed that the filtered Q30 (%) ranged from 94.24 to 94.59% and the GC (%) ranged from 42.59 to 43.15% (Table S3). Unmapped reads were obtained by ribosome matching, and unmapped reads (%) were between 99.66 and 99.84% (Table S4). The alignments with the reference genome showed that the unique mapped (%) ranged from 94.60 to 95.07% (Table S5). Principal component analysis (PCA) can be used to determine duplication between samples and can help to eliminate outliers and improve the accuracy of experimental data. The nine samples in the PCA analysis clearly showed three aggregated distributions in the graph (Figure S1). This means that the sample composition is similar between the biological replicates of the individual treatments, and that the reproducibility between samples is good. The raw transcriptome data are deposited in NCBI under the SRA accession number PRJNA786728.

#### 2.4.1. Analysis of Differentially Expressed Genes (DEGs)

**Figure 2:**
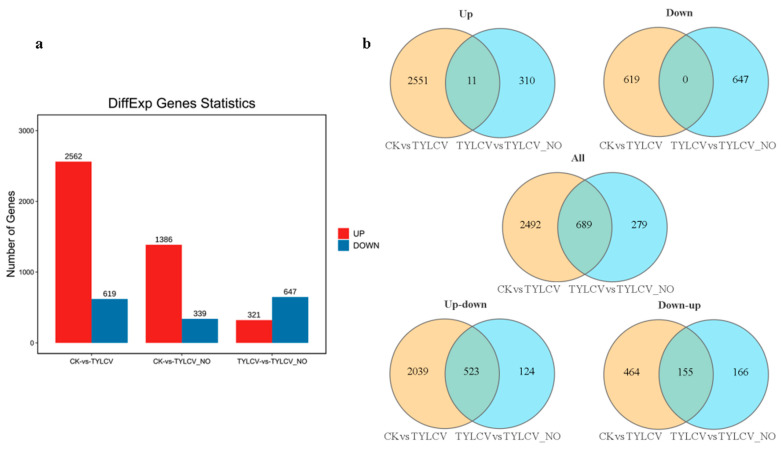
Statistical analysis of DEGs. (**a**) DEGs analysis of tomato responses to exogenous NO and TYLCV at seedling stage. (**b**) Venn diagram of DEGs induced by exogenous NO and TYLCV in tomato seedlings.

A total of 3181 differential genes were screened in CK vs. TYLCV, of which 2562 were up-regulated expression, accounting for 80.54%, and 619 genes were down-regulated expression, accounting for 19.46%. TYLCV vs. TYLCV–NO generated a total of 968 differential genes, of which 321 were up-regulated expression (33.16%) and 647 were down-regulated expression (66.84%) (Figure 2a). This indicates that the up-regulated expression of differential genes predominates in vivo after virus infection in tomatoes, while the down-regulated expression predominates in vivo after exogenous NO induction. There were 689 DEGs in the Venn diagram (Figure 2b), including 523 DEGs that were up-regulated after vaccination and down-regulated after exogenous NO treatment, 155 DEGs that were down-regulated after vaccination and up-regulated after exogenous NO treatment, 11 DEGs that were down-regulated after vaccination and also down-regulated after exogenous NO treatment, and 0 DEGs that were up-regulated after vaccination and also up-regulated after exogenous NO treatment. The number of unique genes induced by exogenous NO was 279 (Figure 2b). This suggests that the main feature of exogenous NO-induced disease is the restoration genes that are abnormally elevated/lowered by the virus while promoting a biological process, and thus resistance is of secondary importance.

#### 2.4.2. Gene Ontology Enrichment Analysis

**Figure 3:**
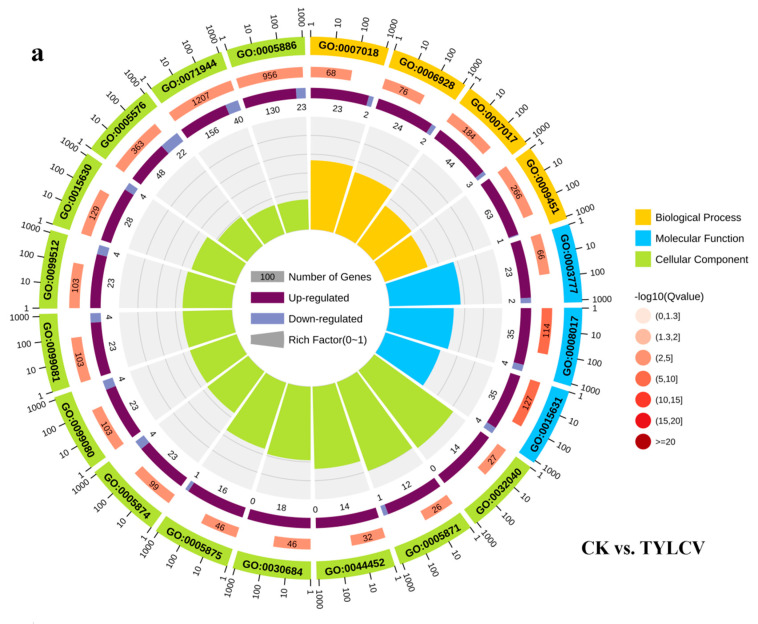

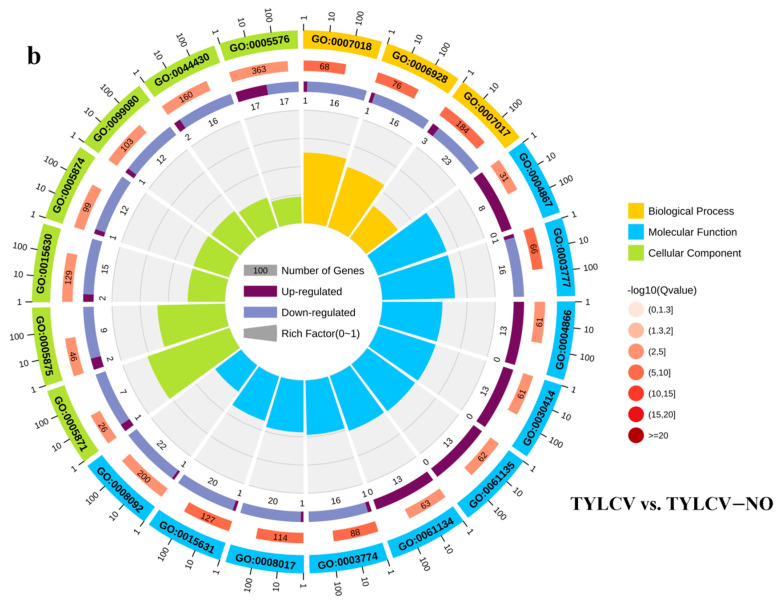
GO enrichment analysis of DEGs in tomato leaf responsive to TYLCV and susceptible tomato responsive to exogenous NO. (**a**) GO enrichment analysis of negative control and positive control producing DEGs. (**b**) GO enrichment analysis of DEGs generated in positive control and treatment groups. The first circle indicates the GO term, and the outside of the circle is the sitting scale of DEGs. Different colors represent different ontologies; the second circle is the number of this GO term in the background of DEGs and the Q-value; the third circle is the bar graph of the proportion of up- and down-regulated differential genes; dark purple represents the proportion of up-regulated DEGs, light purple represents the proportion of down-regulated DEGs; the specific values are shown below; the fourth circle is the RichFactor value of each GO term.

The circle plot results show the top 20 GO terms enriched in the three primary classifications, DEGs in CK vs. TYLCV enriched in the most terms in cellular components, mainly including small-subunit processome, kinesin complexes, preribosome, microtubule-associated complex, microtubule, supramolecular complex, supramolecular fiber, and microtubule cytoskeleton; enriched in biological processes are the next most abundant and include mainly: microtubule-based movement, movement of a cell or subcellular component, and microtubule-based process; molecular functions are the least abundant and include mainly microtubule motor activity, microtubule-binding, and tubulin-binding (Figure 3a). The DEGs in TYLCV vs. TYLCV–NO were enriched in the highest number of molecular functions, mainly serine-type endopeptidase inhibitor activity, microtubule motor activity, endopeptidase inhibitor activity, peptidase inhibitor activity, endopeptidase regulator activity, peptidase regulator activity, motor activity, microtubule binding, tubulin binding, and cytoskeletal protein binding. The next most enriched in cellular components include kinesin complex, microtubule-associated complex, microtubules, and microtubule cytoskeleton, and the least enriched in biological processes include microtubule-based movement, movement of a cell or subcellular component, and microtubule-based process. Interestingly, the terms enriched in CK vs. TYLCV both showed more up-regulation of the DEGs than down-regulation, suggesting that after inoculation of the tomato with the TYLCV, the organism will enter a stressful state and positively regulate various biological processes. Furthermore, all DEGs related to serine-type endopeptidase inhibitor activity, endopeptidase inhibitor activity, peptidase inhibitor activity, endopeptidase regulator activity, and peptidase regulator activity, which were upregulated in TYLCV vs. TYLCV–NO (Figure 3b), suggesting that exogenous NO-induced resistance may be related to the above processes.

#### 2.4.3. KEGG Enrichment Analysis

**Figure 4:**
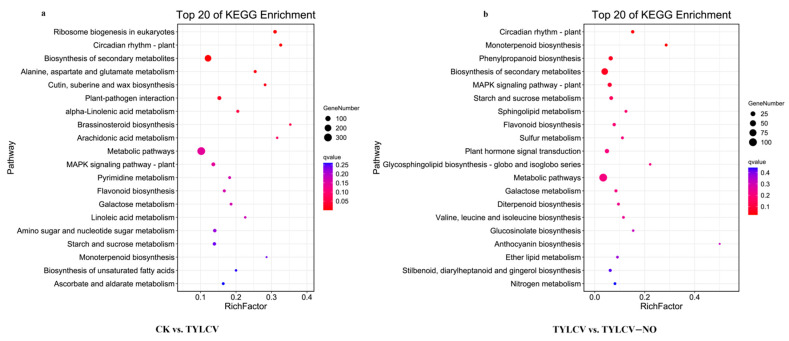
KEGG enrichment analysis of DEGs in tomato leaf responsive to TYLCV and exogenous NO in susceptible tomato. (**a**) KEGG enrichment analysis of negative control and positive control producing DEGs. (**b**) KEGG enrichment analysis of DEGs generated in positive control and treatment groups.

The KEGG enrichment analysis of the DEGs produced by the negative control and positive control groups shows the top 20 pathways with the lowest Q values as shown in Figure 4; the most significant enrichment was found in ribosome biogenesis in eukaryotes; circadian rhythm—plant; biosynthesis of secondary metabolites; “Alanine, aspartate and glutamate metabolism;” cutin, suberin, and wax biosynthesis; plant–pathogen interaction; and another enrichment of DEGs. The most abundant are also metabolic pathways (Figure 4a). The pathways involved in the DEGs produced in the treatment group relative to the positive control group were mainly circadian rhythm—plant; monoterpenoid biosynthesis; phenylpropanoid biosynthesis; biosynthesis of secondary metabolites; MAPK signaling pathway—plant; starch and sucrose metabolism; in addition to pathways enriched for a high number of differential genes, such as metabolic pathways (Figure 4b). 

#### 2.4.4. Regulation of Enzyme Activity-Related Genes and Resistance Proteins by Exogenous NO

Through GO and KEGG enrichment analyses, we identified some of the critical biological processes involved in the regulation of disease resistance in tomatoes by exogenous NO and further analyzed them to find the underlying causes of enhanced resistance in tomatoes. These included enzyme activity, disease-resistance proteins, secondary metabolites, key metabolites, photosynthesis, and peptidase inhibitors.

**Figure 5:**
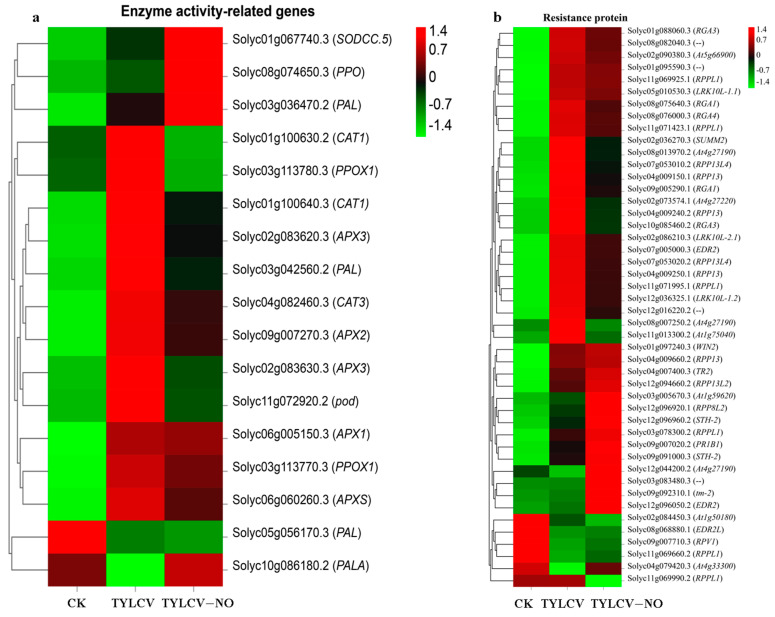
(**a**) Heat map shows the resistance gene expression in tomato. The number of sequenced counts of all genes in the graph is greater than 50; (**b**) Heat map showing expression of genes associated with antioxidant enzyme/defense enzyme in tomato, Different colors indicate the expression level of each gene after normalization by FPKM.2.4.5 exogenous NO-mediated disease resistance proteins.

In this study, the defense enzyme/antioxidant enzyme activities of NO-induced tomato leaves were measured. In order to clarify the mechanism of the exogenous NO regulation of these enzyme activities, we selected genes related to them and performed expression calculations. The results show that the expression levels of *SODCC.5* (Solyc01g067740.3), *PPO* (Solyc08g074650.3), and *PAL* (Solyc03g036470.2) were the highest in the treated group, the second highest in the positive control group, and the lowest in the negative control group. The expression level of *PAL* (Solyc05g056170.3) was highest in the negative control group, second highest in the positive control group, and lowest in the treatment group. *PALA* (Solyc10g086180.2) was expressed at higher levels in the negative control group versus the treatment group than in the positive control group (Figure 5a). The expression levels of all other genes were highest in the positive control group.

Disease resistance proteins play an essential role in plant–pathogen interactions. Tomato disease susceptibility increased the expression levels of most disease-resistance genes, some of which were reduced by exogenous NO induction. In addition to that, another part of the genes was up-regulated and expressed by exogenous NO induction, such as *At1g59620*, *Solyc03g083480.3*, *WIN2*, *RPP13L4*, *TR2*, *RPP13*, *RPPL1*, *At4g33300*, where the *At1g59620* (Solyc03g005670.3), *At4g33300* (Solyc04g079420.3), Solyc03g083480.3, *PR1B1* (Solyc09g007020.2), and *STH-2* (Solyc12g096960.2) expression levels were significantly elevated (Figure 5b). 

#### 2.4.5. Exogenous NO-Mediated Key Secondary Metabolite Genes

**Figure 6:**
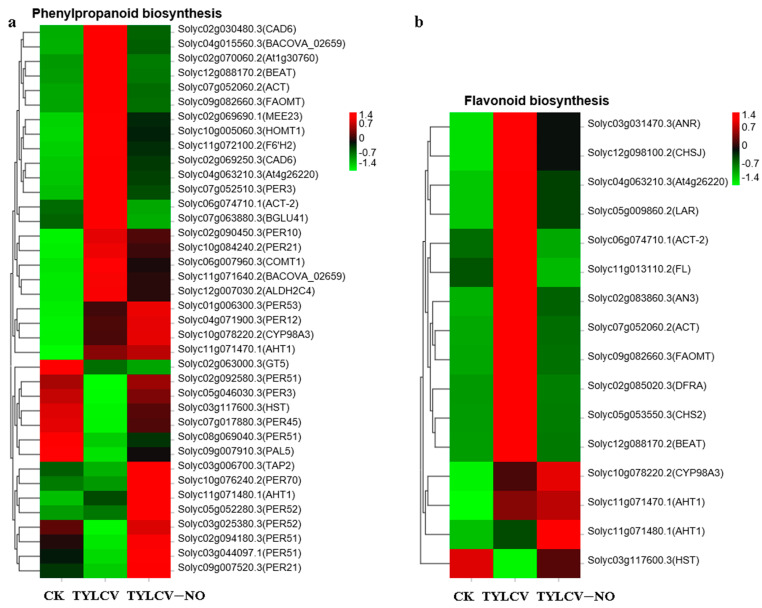
Expression of genes related to secondary metabolites biosynthesis in tomato, (**a**,**b**) are the gene expression of phenylpropanoid biosynthesis and flavonoid biosynthesis, respectively. Different colors indicate the expression level of each gene after normalization by FPKM.

The phenylpropanoid biosynthetic pathway synthesizes numerous resistance substances for plant disease resistance processes. It was found that *PER70* (Solyc10g076240.2), *PER53* (Solyc01g006300.3), *PER52* (Solyc05g052280.3), *PER51* (Solyc02g092580.3), *PER12* (Solyc04g071900.3), *PAL5* (Solyc09g007910.3), and *TAP2* (Solyc03g006700.3) were significantly induced by exogenous NO, and the expression levels of PER70, PER52, and PER51, which are associated with the synthesis of lignin at the end of benzene propane metabolism, were the most elevated (Figure 6a). All the genes related to flavonoid synthesis were up-regulated in tomatoes after TYLCV inoculation except for *HST* (Solyc03g117600.3), while 75% of the genes were down-regulated again after exogenous NO induction, e.g., *At4g26220* (Solyc04g063210.3) and *FL* (Solyc11g013110.2) (Figure 6b). This indicates that at the RNA level, exogenous NO induction restored the abnormal elevation of flavonoid synthesis caused by the TYLCV. 

#### 2.4.6. Exogenous NO-Mediated Key Metabolite Genes

**Figure 7:**
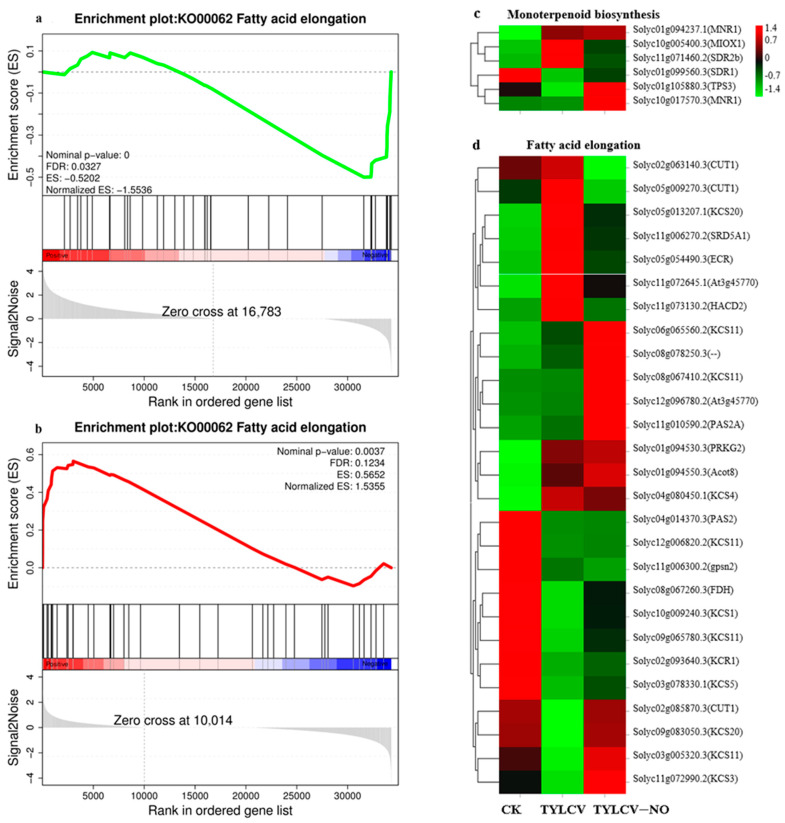
Expression of genes related to the biosynthesis of some key metabolites in tomato, (**a**,**b**) are the GSEA enrichment analysis of all genes generated by CK vs. TYLCV and TYLCV vs. TYLCV–NO, respectively. The expression data of all genes were used to rank the genes using Signal 2 Noise as the criterion. Top: 1 enrichment score (ES) value is calculated for each gene from left to right and is concatenated into a line, with the highest peak being the ES; middle: each black vertical line indicates a gene and its ranked position; bottom: matrix of gene and phenotype associations, with red being highly expressed in the first phenotype and blue being highly expressed in the second phenotype, (**c**,**d**) are the expression profiles of genes related to monoterpene biosynthesis and fatty acid elongation, respectively. Different colors indicate the expression level of each gene after normalization by FPKM.

GSEA enrichment analysis of all sequenced genes revealed that the fatty acid elongation pathway was predominantly down-regulated after viral infection (Figure 7a), while the pathway was up-regulated after exogenous NO induction (Figure 7b). The expression profiles showed that most genes were abnormally elevated or reduced after viral inoculation, and the induction of exogenous NO constrained this abnormal process. A total of 62.96% of the genes was expressed at higher levels than the TYLCV in TYLCV–NO treatment, such as the five *KCS11* (Solyc06g065560.2, Solyc08g067410.2, Solyc12g006820.2, Solyc09g065780.3, Solyc03g005320.3), *PAS2A* (Solyc11g010590.2), *KCS3* (Solyc11g072990.2), *At3g45770* (Solyc12g096780.2), and *CUT1* (Solyc02g085870.3) genes (Figure 7d). Monoterpenoid synthesis is an important process in the plant body against pathogenic bacteria, and in this study, we found that a total of four genes, among which two *MNR1* genes (Solyc01g094237.1, Solyc10g017570.3), *SDR1* (Solyc01g099560.3), and *TPS3* (Solyc01g105880.3), at TYLCV–NO treatment were expressed at higher levels than the TYLCV (Figure 7c).

#### 2.4.7. Effect of Exogenous NO on Photosynthesis

**Figure 8:**
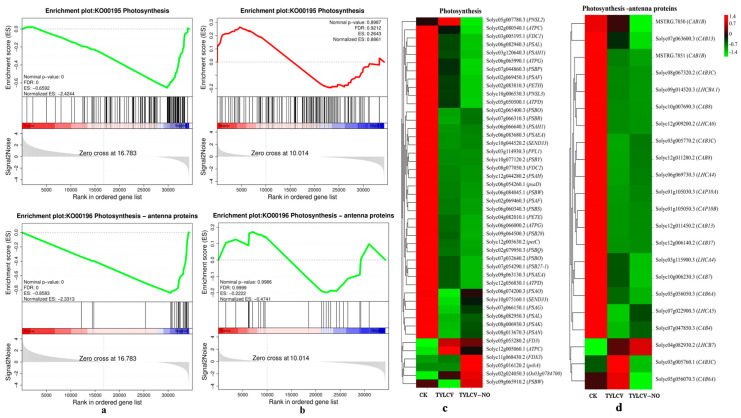
Photosynthesis-related gene expression in tomato, (**a**,**b**) are GSEA enrichment analyses of all genes generated by CK vs. TYLCV and TYLCV vs. TYLCV–NO, respectively, (**c,d**) are the expression profiles of photosynthesis and photosynthesis-antenna protein-related genes, respectively. The number of sequenced counts of all genes in the graph is greater than 50, and different colors indicate the expression level of each gene after normalization by FPKM.

Since no changes in photosynthesis were found by GO with the KEGG enrichment analysis, we performed a GSEA enrichment analysis of all sequenced genes and found that photosynthesis was down-regulated in the positive control group and not significantly found in the treatment group (Figure 8a,b). We further mapped the expression profiles, and overall, the expression levels in the CK treatment were higher than the TYLCV and TYLCV–NO treatments, and 33.33% of the genes were induced by exogenous NO to increase their expression levels compared to the TYLCV treatment, such as *PSBW* (Solyc09g065910.2) and *PSAO* (Solyc06g074200.3) (Figure 8c). The expression of genes in photosynthesis-antennal proteins was similar to that in photosynthesis, involving a total of 22 genes. Among them, the expression levels of 10 genes were induced to be elevated by exogenous NO relative to the TYLCV treatment, such as *CAB4* (Solyc07g047850.3) and *LHCA5* (Solyc07g022900.3) (Figure 8d). The expression of photosynthesis-related genes was down-regulated after the virus infection of the tomato plants, while exogenous NO increased the expression level of down-regulated genes caused by TYLCV, but the increase was not significant.

#### 2.4.8. Exogenous NO-Mediated Peptidase Inhibitors Improve Disease Resistance in Tomato

**Figure 9:**
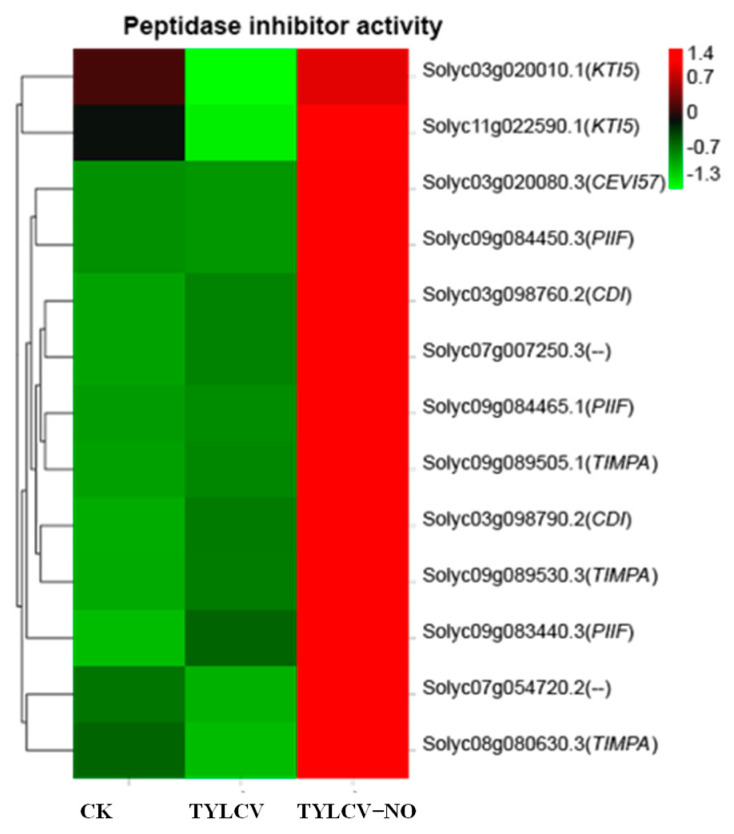
Effect of exogenous NO on peptidase inhibitor-related genes in diseased tomato, different colors indicate the expression level of each gene after normalization by FPKM.

Peptidase inhibitor activity plays an essential role in disease resistance, and according to Figure 9, it can be seen that 46.15% of the peptidase inhibitor-related genes were down-regulated, and 53.85% were up-regulated after virus inoculation in healthy tomatoes. In contrast, after induction by exogenous NO, all peptidase inhibitor-related genes were significantly increased, with *CEVI57*, *TIMPA*, and *PIIF* being the genes with the most significantly elevated gene expression.

#### 2.4.9. qRT-PCR Validation of RNA-Seq with Viral Gene Copy Number Detection

To ensure the reliability of the transcriptome sequencing results, we rigorously selected 20 genes associated with exogenous NO-induced disease resistance in tomatoes and examined their expression levels in CK, TYLCV, and TYLCV–NO treatments. The results show that only 3 genes (15%), namely *MNR1* (Solyc10g017570.3), *PER70* (Solyc10g076240.2), and *pod* (Solyc11g072920.2), did not match with the RNA-Seq results, and the remaining 17 genes (85%) matched the RNA-Seq data perfectly (Table S6). The pod gene, however, matched with POD activity changes. 

### 2.5. Validation of the Mechanism of Exogenous NO Alleviation of Tomato Diseases by Physiological Parameters 

Analysis of differential genes revealed that the virus caused an abnormal elevation or reduction in a large number of genes; however, after exogenous NO treatment, this abnormal elevation or reduction was mainly restored rather than a large number of genes being promoted for expression. Combined with replicative changes in viral genes, it is inferred that the leading cause of symptom relief in diseased plants after exogenous NO treatment is the reduction in viral content caused by the peptidase inhibitor, while the promotion of biological processes associated with disease resistance is a secondary cause. Assuming that the main reason for the increase in resistance is a substantial reduction in virus content, part of the disease resistance response in diseased plants should be attenuated or switched off following exogenous NO treatment. This conclusion has been verified at the transcriptional level. However, changes in transcript levels do not necessarily indicate that the physiological levels are also altered, so we analyzed the physiological indicators. The results of the transcriptome study should be validated at the physiological level. In addition, the replication of the TYLCV in diseased plants should be validated for inhibition to refine the integrity of the final conclusions.

#### 2.5.1. Changes in the Extent of Membrane Lipid Peroxidation and Secondary Metabolites

**Figure 10:**
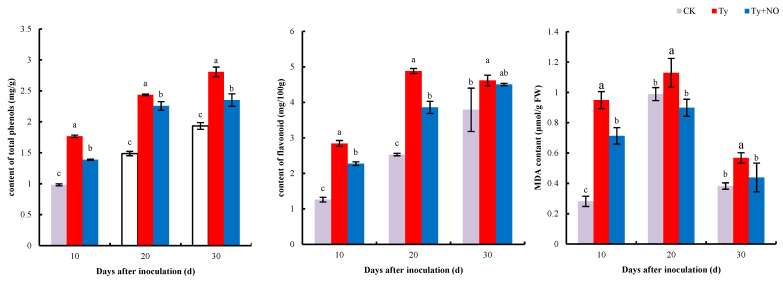
Regulation of total phenols, flavonoids, and MDA content in diseased plants by exogenous NO. Different lowercase letters indicate significant differences between treatments, *p* < 0.05. Same below.

MDA can respond to the degree of membrane lipid peroxidation in plants under adversity. As shown in Figure 10, the MDA content of the tomato was significantly increased (*p* < 0.05) at all three periods after inoculation with the virus, while its content was significantly reduced after spraying with exogenous NO. The total phenol and flavonoid contents were significantly higher in diseased plants compared to healthy plants at all three periods, while the total phenol and flavonoid contents were significantly lower after exogenous NO induction (the difference was not significant at d 30 for flavonoids). The reduction in MDA content ranged from 24.83 to 20.39%, the reduction in total phenols ranged from 21.47 to 7%, and the reduction in flavonoid content ranged from 20.90 to 2.60% under three periods after spraying the diseased plants with exogenous NO.

#### 2.5.2. Changes in the Activity of Antioxidant and Defense Enzymes 

**Figure 11:**
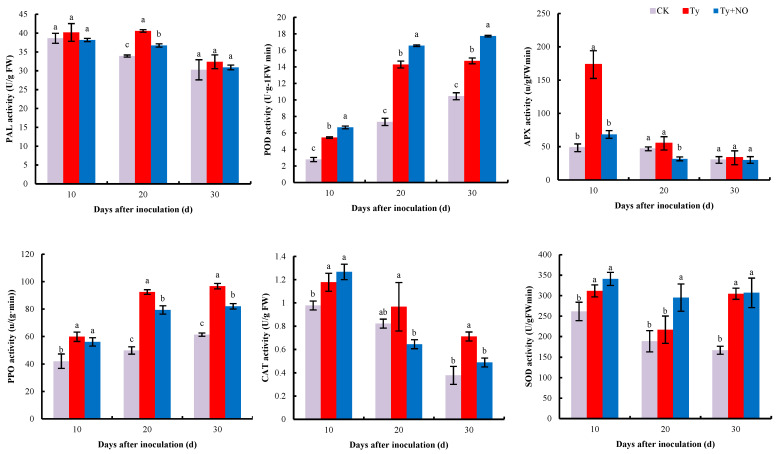
Regulation of antioxidant and defense enzyme activities by exogenous NO in diseased plants. Different lowercase letters indicate significant differences between treatments, *p* < 0.05. Same below.

Exogenous NO had a significant regulatory effect on the physiological parameters of the diseased plants. The activities of PAL, POD, APX, PPO, CAT, and SOD were all induced to varying degrees after inoculation with the virus, with significant differences in POD and PPO activities at all three periods (*p* < 0.05). However, after spraying the diseased plants with exogenous NO, differences emerged in the changes in enzyme activities, with PAL, APX, and PPO activities decreasing at all three periods, POD and SOD activities increasing, and CAT activities increasing at 10 d and decreasing significantly at 20 d and 30 d. The most significant decrease in APX activity was 60.69% at 10 d, and the most significant increase in SOD activity was 36.21% at 20 d after exogenous spraying of NO (Figure 11).

#### 2.5.3. Changes in Photosynthetic Parameters 

**Figure 12:**
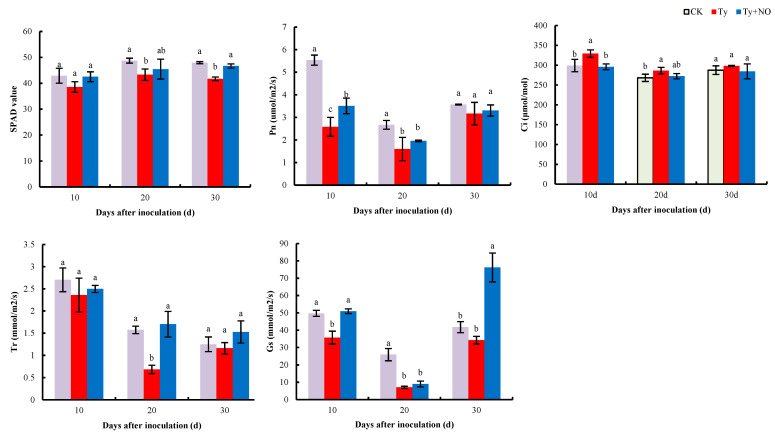
Regulation of chlorophyll content, Pn, Ci, Tr, and Gs in diseased plants by exogenous NO. Different lowercase letters indicate significant differences between treatments, *p* < 0.05. Same below.

We analyzed the photosynthetic parameters to verify whether exogenous NO regulates photosynthesis in diseased plants. The results show (Figure 12) that chlorophyll content, net photosynthetic rate (Pn), transpiration rate (Tr), and stomatal conductance (Gs) showed different degrees of decrease after tomato inoculation with the virus compared to the negative control, while these indicators showed increased characteristics after exogenous NO induction compared to the positive control. In particular, the net photosynthetic rate decreased by 53.39%, 40.23%, and 11.22% in each period, while exogenous NO increased by 35.89%, 22.64%, and 4.36%, respectively. The intercellular CO_2_ concentration (Ci) showed an opposite trend, where at 10 d, Ci was significantly higher by 10.05% in the positive control compared to the negative control but then significantly lower by 10.14% after induction by exogenous NO. This indicates that photosynthesis was improved in diseased plants after the exogenous NO treatment.

### 2.6. Effect of Exogenous NO on the Amplification of TYLCV in Tomato

**Figure 13:**
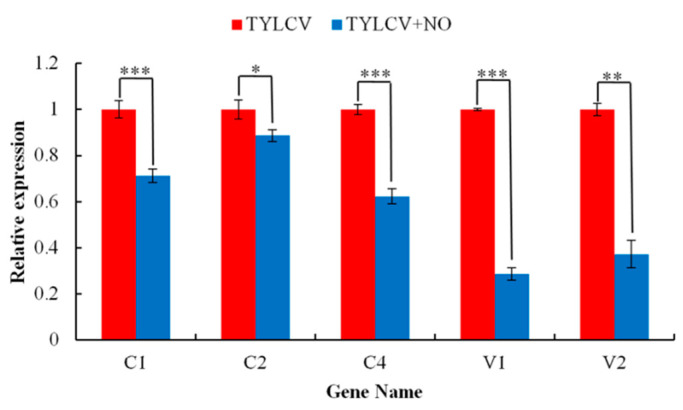
Effect of exogenous NO on the copy number of viral genes. * Means significant difference (*p* < 0.05), ** Means extremely significant difference (*p* < 0.01), *** Means highly significant difference (*p* < 0.001).

To further demonstrate that the main mechanism of exogenous NO-induced resistance is the reduction in viral content triggered by peptidase inhibitors, we further investigated the effect of exogenous NO on the replication of the TYLCV in infected plants. The results show that the copy number of all five major functional genes was reduced after exogenous NO induction, with the expression of the *V2* gene significantly decreased by 71.4% (*p* < 0.001) and the *C2* gene significantly reduced by 11.3% (*p* < 0.05). *C3* was discarded because primers could not be designed (Figure 13).

## 3. Discussion

Tomato is one of the most widely grown vegetable crops worldwide, but the TYLCV threatens the tomato industry in many parts of the world [26]. NO, as a gas-signaling molecule, plays an essential role in plant resistance to stress and, in recent years, has been found to play an important role in inducing disease resistance in plants [27]. As a lipid-soluble free radical signaling substance, NO can regulate the expression of resistance-related genes, activate plant disease resistance defense responses, and also mediate protein nitroxylation/nitrosylation modifications [28]. In addition to this, NO has a complex interaction with key hormones in plant defense, such as SA, JA, and ET that are regulated [29]. The results of Zhou et al. [16] showed that exogenous NO can induce disease resistance in plant fruits. In this study, stem length, root length, dry weight, and fresh weight of tomatoes infected with TYLCV decreased, while stem and root thickness increased. This indicates that longitudinal growth was inhibited, and mainly lateral growth took place in tomatoes infected with the virus. Stem length, root length, root thickness, dry weight, and fresh weight were restored after exogenous NO treatment, while stem thickness increased, indicating that the disease was alleviated and the resistance improved after exogenous NO induction, and that exogenous NO was not able to alleviate the thickening of stems caused by the virus. In addition, this study found no significant difference in dry weight between the treatments, while the fresh weight did differ significantly. The reason for this may be that the TYLCV further influences the water status of the host by affecting the physiological metabolism of tomatoes, while exogenous NO has a positive effect on the maintenance of the water status of the host. Moreover, the virus content was reduced after treatment of tomato plants with exogenous NO. The induction of physiological parameters by exogenous NO mainly showed a tendency to recover toward the control. This suggests that exogenous NO enhanced the growth and tolerance of tomatoes under TYLCV stress and that the reason for the enhancement was related to a reduction in virus content. Meanwhile, our results provide a basis for elucidating the potential pathways of NO-induced disease resistance in tomatoes.

The interaction between plants and pathogens is a complex biological process. The invasion of the TYLCV not only stimulates plants to enter into emergency defense but also causes great damage to seedlings [30]. The production and removal of reactive oxygen species in healthy plants take place in a balanced state, and when the plant is damaged, it causes an outbreak of reactive oxygen species. The excessive accumulation of reactive oxygen species strengthens the peroxidation of membrane lipids, leading to an increase in MDA content and triggering a series of abnormal physiological and biochemical responses, while the presence of antioxidant enzymes can remove the excess reactive oxygen species and bring the plant’s internal environment back into balance [31,32]. The results of this study show that the MDA content and all enzyme activities in tomato increased after the disease, indicating that the plant body entered a state of defense, and the plant resisted the virus invasion by increasing the activities of antioxidant and defense enzymes [20]. The increase in SOD and POD activities and the decrease in MDA content and the rest of the enzyme activities after induction by exogenous NO indicated that membrane lipid peroxidation was reduced and reactive oxygen species levels were restored, which in turn led to a decrease in enzyme activities. However, SOD and POD activities did increase, and transcriptome gene expression verified this change in physiological levels, wherein the *SODCC.5* (Solyc01g067740.3) expression was consistent with the trend in SOD activity. This implies that exogenous NO may indirectly mediate the involvement of SOD and POD in the disease-resistance process of the plant. PAL and PPO are key enzymes in the metabolism of phenylpropane, and enzyme activity was reduced by exogenous NO treatment. Furthermore, this phenylpropane metabolic pathway can form a large number of antimicrobial compounds, such as phenolics and lignin [33]. A total of six disease-resistance-related enzyme activities were examined in this study, four of which showed a decrease after exogenous NO treatment, which already indicates that the imbalanced homeostasis in tomatoes is relieved by exogenous NO treatment and therefore does not require the synthesis of a large number of disease-resistance-related enzymes, and also implies that the main reason for the increase in resistance by exogenous NO is the significant reduction in virus content.

Plants have developed complex immune systems to fight against pathogenic bacteria in order to adapt to their changing and complex living environments [3,34]. The plant’s disease resistance response should therefore be diverse. The presence of a large number of disease-defense proteins in plants is important for inducing systemic resistance in plants and preventing pathogenic bacteria from infesting them [35]. The present study showed that exogenous NO was involved in inducing resistance to the TYLCV in tomato, Solyc03g083480.3, *PR1B1* (Solyc09g007020.2), and *STH-2* (Solyc12g096960.2) in the process of NO-induced disease resistance, and that exogenous NO spraying made tomato more responsive to the virus, while these gene expressions were enhanced perhaps through the enhancement of NO signaling [27]. In addition, 56.52% of the genes were highly expressed by the virus and decreased in the treated group, and 30.43% of the genes were highly expressed by the virus and continued to be expressed in the treated group, *At4g33300* (Solyc04g07942) being the most significant. This suggests that exogenous NO may have altered the tomato’s original disease-resistance system, creating a situation in which restoration and induced resistance coexist.

As one of the three major secondary metabolic pathways, the phenol–propane biosynthetic pathway is closely related to plant disease resistance and signaling, and its metabolites include flavonoids, terpenoids, lignans, etc. [36]. In this study, exogenous NO induced the expression levels of genes related to the biosynthesis of phenylpropanoids so that their terminal lignin synthesis-related genes (*PER70, PER51,* and *PER52*) were up-regulated, in addition to *TAP2, PAL5, PER53*, and *PER12*. A total of 75% of the genes involved in flavonoid synthesis were down-regulated, and the validation study in the physiological section of this paper found that flavonoid levels were also reduced in the treatment group. The total phenolic content was also reduced. This again demonstrates that the spraying of exogenous NO resulted in a substantial reduction in the degree of damage to the diseased plant and therefore a weakening of the synthesis process of total phenols and flavonoids, and again implies that the main reason for the enhancement of resistance by exogenous NO was a substantial reduction in virus content. Exogenous NO may regulate the synthesis of lignin involved in the process of induced resistance. Fatty acids and their derivatives are involved in PTI, SAR, and R gene-mediated ETI in plant disease resistance defense. Long-chain fatty acids are components of waxes, keratin, and cork, and are thus involved in plant defense against pathogenic bacteria [37]. Studies have shown that sixteen- and eighteen-carbon fatty acids are involved in plant disease resistance and microbial basal defense; for example, the accumulation of 16:1, 18:1, and 16:3 fatty acids can enhance the resistance to yellow wilt [38]. In this study, 62.96% of the fatty acid-related genes were found to be induced by exogenous NO to increase their expression levels, especially *At3g45770, KCS11, CUT1*, and *KCS3*. It is suggested that exogenous NO promotes the elongation of fatty acids, which are mostly long-chain fatty acids associated with plant disease resistance, and exogenous NO induces disease resistance in tomato by regulating fatty acid elongation. Monoterpenes are widely found in the plant world and are closely related to plant resistance, and terpenes have the ability to inhibit the growth of pathogenic bacteria and transmit disease-resistance signals [39]. For example, the proportion of limonene was significantly higher in highly resistant asexual lines than in susceptible asexual lines in the interaction between Pinus sylvestris and nematodes [40]. In this study, the terpene synthesis of MNR1 and TPS3 genes was significantly induced by exogenous NO with elevated expression levels and involved in terpenoid synthesis, and the exogenous NO-induced monoterpenoid synthesis improved the resistance to the TYLCV in tomatoes. The evolutionary history of plants is quite long and must rely on multiple pathways in disease-resistance defense rather than a single one. NO is again an important signaling molecule in plants, so exogenous NO also indirectly regulates multiple pathways, meaning that even though the viral content of the diseased plant is reduced, the plant will still run some disease resistance programs, such as continued synthesis of disease-resistance proteins, increased activity of some enzymes, long-chain fatty acids, and synthesis of monoterpenes.

Photosynthesis is involved in various life activities as the power system of plants, and the multiplication of viruses in the host can hinder its normal physiological and metabolic processes [41]. It has been shown that most plants infected with viruses have increased chlorophyllolytic enzyme activity, which converts chlorophyll into chlorophyll lipids and chlorophyll alcohols, causing a decrease in chlorophyll content, affecting photosynthetic electron transfer activity and reducing photosynthesis in the host plant [42]. In the present study, the photosynthetic parameters of the tomato showed different degrees of drastic decreases after vaccination for the disease, and only the intercellular CO_2_ concentration increased, indicating that the phenomenon of a virus-induced decrease in photosynthesis was caused by non-stomatal factors, which is consistent with the findings of Yu Li et al. [43]. After exogenous NO induction, the chlorophyll content increased and each photosynthetic index showed different degrees of recovery, but the degree of recovery was not significant, and this result was verified at the transcriptional level. The transcriptome results show that genes related to photosynthetic and photosynthetic-antenna proteins were generally all down-regulated after tomato infection with the virus, and only a few of these down-regulated genes were slightly up-regulated in expression after exogenous NO induction. In addition, exogenous NO significantly induced the expression of *PSBW* and *PSAO* in photosynthesis with *CAB4* and *LHCA5* in photosynthetic-antenna proteins, which may be associated with elevated photosynthesis in the treatment group. This indicates that the virus severely damaged photosynthesis in tomatoes, and even though this damage was difficult to repair completely by exogenous NO, the treatment with exogenous NO still improved photosynthesis in the diseased plant, demonstrating that exogenous NO did improve disease tolerance in tomatoes.

Ribosome biogenesis in eukaryotes was significantly enriched after inoculation with the TYLCV but not in “TYLCV vs. TYLCV-NO.” This suggests that the invasion of the TYLCV may affect this biological process and thus protein production, and that the main reason why exogenous NO increases resistance in tomatoes may not be related to this process. The above analysis suggests that the reduction in virus content after exogenous NO treatment is the main reason for the increased resistance in tomatoes, but the main mechanism for the reduction in virus content is still unknown. Studies have shown that the uncoupling of microtubules impedes HR, exacerbates pathogen invasion, and reduces plant disease resistance [44]. Protease inhibitors are involved in the defense of plants against pathogenic microorganisms; a large number of proteins used in the replication of biphasic viruses are obtained from host plants; thus, these proteases are necessary for the invasion of plant cells and the provision of nutrients and plant SPIs are able to inhibit extracellular serine proteases produced by plant pathogenic microorganisms. It has been shown that the up-regulation of SPi-encoding genes enhances plant defenses against biotic stresses [45]. GO enrichment analysis revealed that microtubule-like biological activities were upregulated after tomato vaccination, whereas they were all down-regulated in the treatment group. Serine-type endopeptidase inhibitor activity, endopeptidase inhibitor activity, peptidase inhibitor activity, endopeptidase regulator activity, and peptidase regulator activity were all induced by exogenous NO, thereby inhibiting protein hydrolysis. TYLCV encodes a total of six ORFs (*C1, C2, C3, C4, V1,* and *V2*), of which *C1* encodes a replication-associated protein (Rep), *C2* encodes a transcriptional activator protein (TrAP), *C3* encodes a replication-enhancing protein (REn), the *C4* gene is involved in the pathogenicity of the TYLCV (Rep), *V1* encodes a coat protein (*CP*), V2 is associated with viral movement and inhibition of host immune response [46], and the proteins required for the synthesis of the capsid protein account for the major part of the viral replication. The expression of the *V1* gene-encoding shell protein decreased the most after exogenous NO-induced resistance, by 71.4%, indicating that exogenous NO was the first to disrupt the synthesis of the viral shell protein by inducing various peptidase inhibitors to inhibit protein hydrolysis, resulting in a reduction in protein synthesis raw material, which eventually blocked virus replication and caused the recovery of infected tomato plants. This is similar to the antiviral mechanism in animals, in which antiviral studies are usually performed by genetic means to increase the peptidase inhibitor activity to achieve the antiviral goal. Interestingly, no exogenous NO-induced peptidase inhibitors have been reported [47]. Overall, exogenous NO improved disease resistance in tomatoes by regulating peptidase inhibitors, gene expression, monoterpenes, and lignans mainly in terms of blocking virus capsid protein synthesis, slowing virus replication and movement, and relieving the virus inhibition of host immune responses. However, all DEGs for peptidase inhibitors were up-regulated, presumably being the main reason for the reduced viral content in diseased plants and for the increased resistance in tomatoes.

## 4. Materials and Methods

### 4.1. Experimental Materials and Virus Inoculation

The tomato material tested was the recognized susceptible cultivar Jinpeng 1 [48]. The TY-DNA infectious clone pBin-PLUS-1.7A + 2β (Agrobacterium tumefaciens) used for seedling infection was obtained from the laboratory of Prof. Xueping Zhou at Zhejiang University. Before inoculation, Agrobacterium containing TYLCV-[CN: SH2] was coated in solid LB medium and incubated in a dark incubator at 28 °C in an inverted position, followed by access to liquid LB medium in a shaker at 200 r/min 28 °C overnight. Both solid and liquid medium contained Kan (50 mg/L) and Rif (50 mg/L), and the OD_600_ of the bacterial solution reached 0.7 at the time of the inoculation experiment. Excessive watering the night before virus inoculation was performed to allow for the successful invasion of TYLCV. Tomato plants of uniform growth at 30 d after sowing were selected and the bacterial solution was slowly injected into the leaves of seedlings using a syringe at 0.3 mL per plant [49]. 

### 4.2. Experimental Treatments 

The seeds were soaked in 10% NaClO solution for 10min and then washed 3 times in distilled water. The full seeds were selected and cultured in 32-well seedling trays and transplanted into pots (22 cm diameter, 17 cm high), and after 1–2 true leaves had grown. Growing conditions were: room temperature 25 °C, light intensity about 450 μmol-m^−2^-s^−1^, and pot positions changed every 3 days to ensure consistent light. Three treatments (CK, negative control, no treatment; TYLCV, positive control, inoculated with the virus; TYLCV–NO, inoculated with virus and sprayed with SNP solution at a concentration of 0.15 mmol/L) were set up 30 d after sowing, and three periods (10, 20, and 30 d after virus inoculation) were set for each treatment. The concentrations used for SNP were derived from previous studies in our laboratory and the SNP solution contained 0.1% Tween 20 to promote the uptake of the spray solution by the plants. The SNP solution was sprayed three times on three consecutive evenings prior to virus inoculation, once each day at 30 mL per plant, and once more on the evening of the same day, at 10 and 20 d after virus inoculation, when index measurements and sampling were completed, meaning a total of five sprays per plant. Morphological counts and surveys were carried out at 10 d, 20 d, and 30 d after virus inoculation, and healthy leaves were selected from the same location on each plant for sampling. A total of 10 g of samples were taken from all plants under each treatment, samples were mixed and divided into 20 portions and stored (−80 °C) for use in subsequent experiments. 

### 4.3. Virus PCR Detection

On the 9th and 36th d after inoculation, tomato leaves were taken and the total DNA of the leaves was extracted with the kit (Plant Genomic DNA Kit, Tiangen Biochemical Technology Ltd., Beijing, China). The primer used was CP (F: TGTTCCCCGTGGATGTGAAG; R: GTTCTCGTACTTGGCTGCCT) with an amplification length of 478 bp. The PCR system was 20 µL, including 1 µL of template DNA, 10 µL of Master Mix, 1 µL of upstream primer, 1 µL of downstream primer, and 7 µL of ddH_2_O. The reaction conditions were as follows: denaturation at 95 °C for 5 min and then starting the cycle, denaturation at 95 °C for 30 s, annealing at 61 °C for 30 s, extension at 72 °C for 1 min, and after 30 cycles, continue extension at 72 °C for 7 min and store at 4 °C. PCR products were electrophoresed on a 1.0% agarose gel for 25 min (150 V) and finally stained with Goldview and photographed with a gel imaging system [50]. 

### 4.4. Measurement of Phenotypic Indicators and Analysis of Disease Severity

Referring to the method of Lapidott et al. [51], plant height, root length, stem root thickness, and biomass were measured at 30 d after inoculation, and the plants were separated from the above-ground parts from the cotyledonary parts, killed at 105 °C for 30 min, dried at 80 °C to constant weight, and then weighed. The disease was investigated and counted at the 30th d of infection, and tomato plants infected with TYLCV were classified into four classes according to the severity of the disease: 0 = no disease, 1 = slight yellowing and curling of leaf edges, 2 = leaves exhibiting moderate yellowing and curling, 3 = severe yellowing and curling of leaves, and 4 = severe curling and yellowing of leaves along with smaller leaves and dwarfing of plants.

Infection rate (%) = (number of infected plants/total number of plants) × 100%

Disease severity (%) = (Σ(disease level × number of plants at each disease level)/(4 × total number of plants)) × 100%

Relative control effect (%) = ((control disease severity − treatment disease severity)/disease severity of control) × 100%

### 4.5. Detection of Physiological Indices

The leaves of the same part of each plant were selected, and the relative chlorophyll content (SPAD value) was determined by using the TYS-B chlorophyll meter to obtain the average value. Net photosynthetic rate (Pn), intercellular CO_2_ concentration (Ci), stomatal conductance (Gs), and transpiration rate (Tr) were measured between 9:00 and 11:00 a.m. using a handheld photosynthesiometer CI-340. The enzyme solution extraction process is as follows: weigh 0.5 g tomato leaves and put them into a mortar, then add 5 mL PBS with PH = 7.8, grind in an ice bath, pour homogenate into a centrifugal tube, freeze centrifugation for 20 min (10,000× *g*), pour supernatant (enzyme liquid) into a test tube, and store at 0–4 °C for future use [52]. For the determination of SOD activity, refer to the NBT photochemical reduction method of K. Chahid et al. [53]. The steps for the determination of CAT and POD activities were referred to in the method of Yang et al. with slight modifications [54]. For the determination of APX activity: use the UV absorption method [55]. The measurement of MDA content (malondialdehyde) was based on the method of Heath R. et al. with slight modifications [56]. Phenylalanine amylolytic enzyme activity (PAL) was determined by analyzing the content of cinnamic acid [57]. The determination of polyphenol oxidase (PPO) activity was performed by the colorimetric method with slight modifications referring to Spagna et al. [58]. The total phenolic and flavonoid contents were determined by the Folin–Ciocalteu method [59] and the aluminum nitrate colorimetric method [60], respectively, and gallic acid and rutin were used as standards. Three replicates were set up. The biochemical parameters were measured uniformly after all 3 sampling sessions were completed.

### 4.6. RNA Extraction, Library Construction, and Sequencing

Nine tomato samples were selected on the 20th day after inoculation, three samples from each treatment and total RNA were extracted from the leaves using the Trizol kit method; refer to the instructions in the kit for extraction methods. RNA quality was assessed on an Agilent 2100 (Agilent Technologies, Palo Alto, CA, USA), while agarose gel electrophoresis was performed to check its integrity. After passing RNA quality control, mRNA was enriched with Oligo (dT) beads, while unwanted rRNA was removed using the Ribo-ZeroTM Magnetic Kit (Epicentre, Madison, WI, USA). Short fragments of mRNA were obtained using a fragmentation buffer, and cDNA was obtained by adding random primers through reverse transcription experiments. The obtained cDNA was added to DNA polymerase I, RNase H, dNTP, and buffer to synthesize second-strand cDNA. Finally, cDNA fragments were purified using the QiaQuick PCR extraction kit (Qiagen, Venlo, The Netherlands), end-repaired, A bases added, and adapters ligated. Once again, agarose gel electrophoresis was performed to select the size of the ligated products, PCR amplification, and sequencing using Illumina Novaseq6000 (Gene Denovo Biotechnology Co., Guangzhou, China).

### 4.7. RNA-Seq Data Analysis

The raw data generated from the sequencing of the nine constructed libraries under the 3 treatments of CK, TYLCV, and TYLCV–NO were subjected to quality control, ribosome database matching, and reference genome matching before the FPKM (fragments per kilobase million) method was used to calculate the gene expression level of each sample. Differential gene expression analysis was performed using DESeq2 [61] software, and differentially expressed genes were obtained using FDR < 0.05 and |log2FC| > 1 as screening conditions. The steps are as follows: quality control the raw reads obtained by the sequencer with fastp [62] to obtain clean reads, and then match the clean reads to the species ribosome database with the short-reads, matching tool bowtie2 [63] to remove the reads matched to the ribosome without allowing for mismatches. Reference genome (ensemble bl_release51)-based alignment analysis was performed using HISAT2 [64] software and transcripts were reconstructed using the Stringtie method [65]. Principal component analysis (PCA) was carried out using R (http://www.r-project.org/ (accessed on 13 July 2021)) based on gene expression data.

### 4.8. GO and KEGG Enrichment Analysis

First, we mapped the filtered DEGs to each term of the GO [66] database (http://www.geneontology.org/ (accessed on 13 July 2021)) and counted the number of differential genes in each term, and then applied a hypergeometric test to identify the significantly enriched terms. The KEGG [67] is the major public path-related database. A GO-like approach was applied to identify the pathway in which the differential genes were significantly enriched, so as to identify the most significant biological pathway in which the differential genes were involved, with corrected *p* value ≤ 0.05 as the significant enrichment condition. The GSEA [68] analysis can compensate for problems such as insufficient effective information mining for low expression levels by traditional enrichment analysis and a more comprehensive interpretation of a functional unit. The expression data of all genes are ranked by signal-to-noise ratio, which is calculated as (μa − μb)/(σa + σb), wherein μ is the mean, σ is the standard deviation, and both a and b represent groupings. The gene set is analyzed for whether it is more or less advanced in the ranking of all genes, and then the pathway or GOterm in which the gene set is located is scored to obtain an ES (enrichment score) value. A permutation test was performed based on the gene set, significance *p*-values were calculated, and finally, the normalized ES values (NES values) were corrected for multiple tests to obtain the FDR values. We believe that the gene collections under pathways with |NES| > 1, nominal *p* value (NOM *p*-value) < 0.05, and false discovery rate (FDR) q-value < 0.25 are meaningful.

### 4.9. qRT-PCR Analysis

To further verify the accuracy of the sequencing data, we selected 20 representative differentially expressed genes for qRT-PCR analysis, with reference to UBI screened by Jiang et al. [69], under tomato TYLCV treatment as the internal reference gene. The primer sequence information is listed in Table S1. Validation test using RNA samples used for transcriptome sequencing. Firstly, cDNA was synthesized by reverse transcription using SweScript I First Strand cDNA Synthesis Kit (Wuhan, China); secondly, amplification experiments were performed using SYBR Green Premix Pro Taq HS qPCR Kit (Wuhan, China), and finally, the 2^−ΔΔCT^ method was used to calculate the relative gene expression levels [70]. The experiment was repeated three times for each sample.

### 4.10. Statistical Analysis of Data

The data obtained from the experiments were analyzed using SPSS 20.0 statistical software (IBM Corporation, Armonk, NY, USA), and significance analysis was performed using one-way analysis of variance (ANOVA), and we considered the difference to be significant at *p* < 0.05 in Duncan’s test.

## 5. Conclusions

**Figure 14:**
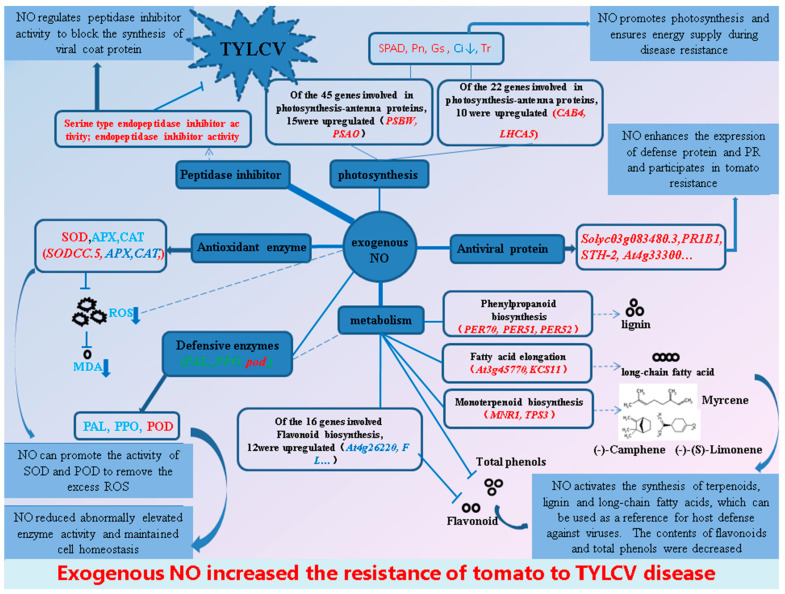
Exogenous NO-mediated resistance of tomato seedlings to TYLCV based on transcriptome and physiological changes. Up-regulated and down-regulated genes are represented in red and green, respectively, and up-regulated genes are represented in blue. The thickness of the line connected to the central circle indicates the degree of response of the connected function to the exogenous NO.

The results of the study show that both the above- and below-ground parts of tomatoes infected with the TYLCV induced by exogenous NO recovered to different degrees, mainly in the form of increased plant height and fresh weight, with a relative control effect of 44.0%. Exogenous NO enhanced the tomato resistance to the TYLCV and reduced virus content in plants by improving antioxidant enzyme (SOD, POD) activity, resistance protein synthesis, lignin content, long-chain fatty acid content, terpene content, peptidase inhibitor activity, and photosynthesis. However, peptidase inhibitors are the main reason for the enhanced resistance of tomatoes. In contrast, most enzyme activities, as well as total phenol and flavonoid contents, exhibited a decreasing trend (Figure 14), which is further evidence that the reduction in viral content caused by the peptidase inhibitor was the main reason for the increased resistance, as the significant reduction in viral content caused some of the resistance processes to be weakened or turned off. Overall, the present study contributes to further understanding the mechanism of exogenous NO-mediated resistance to the TYLCV in tomatoes from the RNA level.

## Figures and Tables

**Table 1:** Effects of exogenous NO on biomass and stem/root diameter of susceptible tomato.

Treatments	Dry Weight (g)	Fresh Weight (g)	Stem Diameter (mm)	Root Diameter (mm)
CK	2.60 ± 0.44 ^a^	34.00 ± 1.31 ^b^	4.71 ± 0.30 ^b^	2.12 ± 0.58 ^b^
TYLCV	2.17 ± 0.45 ^a^	23.97 ± 1.67 ^c^	5.23 ± 0.16 ^a^	3.56 ± 0.51 ^a^
TYLCV + NO	2.93 ± 0.65 ^a^	37.13 ± 1.60 ^a^	5.25 ± 0.23 ^a^	3.20 ± 0.55 ^ab^

Note: Different lowercase letters indicate significant differences under different treatments, *p* < 0.05.

**Table 2:** Prevention and treatment effects of exogenous NO on TYLCV.

Treatments	Total Number of Plants	Number of Plants Infected by TYLCV	Infection Rate (%)	Disease Index (%)	Control Rate (%)
CK	20	0	0%	—	—
TYLCV	20	20	100%	62.5%	—
TYLCV + NO	20	20	100%	35.0%	44.0%

**Table 3:** Results of RNA sample quality detection.

Sample Name	Concentration (ng/μL)	Volume (μL)	Total (μg)	RIN Value
CK-1	612	40	24.48	7.7
CK-2	486	39	18.95	7.8
CK-3	585	38	22.23	7.4
TYLCV-1	495	38	18.81	7.6
TYLCV-2	621	39	24.22	7.5
TYLCV-3	486	38	18.47	7.5
TYLCV-NO-1	591	39	23.05	7.6
TYLCV-NO-2	573	38	21.77	7.5
TYLCV-NO-3	462	38	17.56	7.6

## Data Availability

Data are contained within the article or Supplementary Materials.

## Supplementary Materials

The following supporting information can be downloaded at: https://www.mdpi.com/article/10.3390/ijms232012542/s1.

## References

[1] Ahmad P., Ahanger M., Alyemeni M., Wijaya L., Alam P. (2018). Exogenous application of nitric oxide modulates osmolyte metabolism, antioxidants, enzymes of ascorbate-glutathione cycle and promotes growth under cadmium stress in tomato. Protoplasma.

[2] Dhaliwal M.S., Jindal S.K., Sharma A., Prasanna H.C. (2020). Tomato yellow leaf curl virus disease of tomato and its management through resistance breeding: A review. J. Hortic. Sci. Biotechnol..

[3] Scholthof K.B., Adkins S., Czosnek H., Palukaitis P., Jacquot E., Hohn T., Hohn B., Saunders K., Candresse T., Ahlquist P. (2011). Top 10 plant viruses in molecular plant pathology. Mol. Plant Pathol..

[4] Riley D., Srinivasan R. (2019). Integrated Management of Tomato Yellow Leaf Curl Virus and its Whitefly Vector in Tomato. J. Econ. Entomol..

[5] Sade D., Sade N., Brotman Y., Czosnek H. (2020). Tomato yellow leaf curl virus (TYLCV)-resistant tomatoes share molecular mechanisms sustaining resistance with their wild progenitor Solanum habrochaites but not with TYLCV-susceptible tomatoes. Plant Sci..

[6] Dasgupta A., Sinha S.K., Praveen S. (2004). Structure of replication initiator protein unites diverse viruses causing tomato leaf curl disease (ToLCD). Plant Sci..

[7] Hormuzdi S.G., Bisaro D.M. (1995). Genetic analysis of beet curly top virus: Examination of the roles of L2 and L3 genes in viral pathogenesis. Virology.

[8] Kong L.J. (2000). A geminivirus replication protein interacts with the retinoblastoma protein through a novel domain to determine symptoms and tissue specificity of infection in plants. EMBO J..

[9] Mati S., Pegoraro M., Noris E. (2016). The C2 protein of tomato yellow leaf curl Sardinia virus acts as a pathogenicity determinant and a 16-amino acid domain is responsible for inducing a hypersensitive response in plants. Virus Res..

[10] Selth L.A., Dogra S.C., Rash Eed M.S., Randles J.W., Rezaian M.A. (2006). Identification and Characterization of a Host Reversibly Glycosylated Peptide that Interacts with the Tomato leaf curl virus V1 Protein. Plant Mol. Biol..

[11] Deng Y., He S., Geng Q., Duan Y., Guo M., Li J., Cao Y. (2015). Synthesis and biological activity evaluation of novel amino acid derivatives as potential elicitors against Tomato yellow leaf curl virus. Amino Acids.

[12] Lapidot M., Friedmann M., Lachman O., Yehezkel A., Nahon S., Cohen S., Pilowsky M. (1997). Comparison of Resistance Level to Tomato Yellow Leaf Curl Virus Among Commercial Cultivars and Breeding Lines. Plant Dis..

[13] Ren Y., He J., Liu H., Liu G., Ren X. (2017). Nitric oxide alleviates deterioration and preserves antioxidant properties in ‘Tainong’ mango fruit during ripening. Hortic. Environ. Biotechnol..

[14] Del C.F., Nejamkin A., Cassia R., Correa-Aragunde N., Fernández B., Foresi N., Lombardo C., Ramirez L., Lamattina L. (2019). The era of nitric oxide in plant biology: Twenty years tying up loose ends. Nitric Oxide Biol. Chem..

[15] Hong J.K., Yun B.W., Kang J.G., Raja M.U., Kwon E., Sorhagen K., Chu C., Wang Y., Loake G.J. (2008). Nitric oxide function and signalling in plant disease resistance. J. Exp. Bot..

[16] Zhou Y., Li S., Zeng K. (2016). Exogenous nitric oxide-induced postharvest disease resistance in citrus fruit to Colletotrichum gloeosporioides. J. Sci. Food Agr..

[17] Lu R., Liu Z., Shao Y., Su J., Li X., Sun F., Zhang Y., Li S., Zhang Y., Cui J. (2020). Nitric Oxide Enhances Rice Resistance to Rice Black-Streaked Dwarf Virus Infection. Rice.

[18] Liu L., Yu M., Zheng Y., Sheng J., Shen L. (2010). Effect of Postharvest nitric oxide treatment on jasmonates biosynthesis and disease incidence to botrytis cinerea pers. in tomato fruits. Food Sci..

[19] Kobeasy M.I., El-Beltagi H.S., El-Shazly M.A., Khattab E.A.H. (2011). Induction of resistance in *Arachis hypogaea L.* against Peanut mottle virus by nitric oxide and salicylic acid. Physiol. Mol. Plant Pathol..

[20] Hermes V.S., Asta P.D., Amaral F.P., Anacleto K.B. (2013). The regulation of transcription of genes related to oxidative stress and glutathione synthesis in Zea mays leaves by nitric oxide. Biol. Plantarum..

[21] Kelm M., Dahmann R., Wink D., Feelisch M. (1997). The nitric oxide/superoxide assay. Insights into the biological chemistry of the NO/O-2. interaction. J. Biol. Chem..

[22] Gao W.Z. (2021). Study on Identification Method of Tomato Chlorosis Virus Disease and Preliminary Mapping of Resistance Genes. Master’s Thesis.

[23] Jing C., Zhang H., Feng M., Zuo D., Jiang T. (2016). Transcriptome analysis of woodland strawberry (Fragaria vesca) response to the infection by Strawberry vein banding virus (SVBV). Virol. J..

[24] Chen T., Lv Y., Zhao T., Li N., Yang Y., Yu W., He X., Liu T., Zhang B. (2013). Comparative transcriptome profiling of a resistant vs. susceptible tomato (*Solanum lycopersicum*) cultivar in response to infection by tomato yellow leaf curl virus. PLoS ONE.

[25] Coppola V., Coppola M., Rocco M., Digilio M., D’Ambrosio C., Renzone G., Martinelli R., Scaloni A., Pennacchio F., Rao R. (2013). Transcriptomic and proteomic analysis of a compatible tomato-aphid interaction reveals a predominant salicylic acid-dependent plant response. BMC Genom..

[26] Rabie M., Ratti C., Abdel Aleem E., Fattouh F. (2017). Detection and molecular characterization of tomato yellow leaf curl virus naturally infecting *Lycopersicon esculentum* in Egypt. Acta Virol..

[27] Agurla S., Gayatri G., Raghavendra A.S. (2014). Nitric oxide as a secondary messenger during stomatal closure as a part of plant immunity response against pathogens. Nitric Oxide.

[28] Bellin D., Asai S., De Lledonne M., Yoshioka H. (2013). Nitric Oxide as a Mediator for Defense Responses. Mol. Plant Microbe. Interact..

[29] Wendehenne D., Durner J., Klessig D.F. (2004). Nitric oxide: A new player in plant signalling and defence responses. Curr. Opin. Plant Biol..

[30] Seo J., Kim M., Kwak H., Choi H., Nam M., Choe J., Choi B., Han S., Kang J., Jung C. (2018). Molecular dissection of distinct symptoms induced by tomato chlorosis virus and tomato yellow leaf curl virus based on comparative transcriptome analysis. Virology.

[31] Jaiswal N., Singh M., Dubey R., Venkataramanappa V., Datta D. (2013). Phytochemicals and antioxidative enzymes defence mechanism on occurrence of yellow vein mosaic disease of pumpkin (*Cucurbita moschata*). 3 Biotechnol..

[32] Li T., Wang Y., Huang Y., Liu J., Xing G., Sun S., Li S., Xu Z., Xiong A. (2020). A novel plant protein-disulfide isomerase participates in resistance response against the TYLCV in tomato. Planta.

[33] Maddox C.E., Laur L.M., Tian L. (2010). Antibacterial activity of phenolic compounds against the phytopathogen Xylella fastidiosa. Curr. Microbiol..

[34] Ali S., Ganai B., Kamili A., Bhat A., Mir Z., Bhat J., Tyagi A., Islam S., Mushtaq M., Yadav P. (2018). Pathogenesis-related proteins and peptides as promising tools for engineering plants with multiple stress tolerance. Microbiol. Res..

[35] Li X.Y., Gao L., Zhang W.H., Liu J.K., Liu D.Q. (2015). Characteristic expression of wheat PR5 gene in response to infection by the leaf rust pathogen, Puccinia triticina. J. Plant Interact..

[36] Ahuja I., Kissen R., Bones A.M. (2012). Phytoalexins in defense against pathogens. Trends Plant Sci..

[37] Khanal B.P., Knoche M. (2017). Mechanical properties of cuticles and their primary determinants. J. Exp. Bot..

[38] Xing J., Chin C.K. (2000). Modification of fatty acids in eggplant affects its resistance to Verticilliumdahliae. Physiol. Mol. Plant Pathol..

[39] Duccio R.L., Caccioni A., Monica Guizzardi A., Daniela M., Biondi B., Agatino Renda B., Giuseppe Ruberto B. (1998). Relationship between volatile components of citrus fruit essential oils and antimicrobial action on Penicillium digitatum and Penicillium italicum. Int. J. Food Microbiol..

[40] Wei Y. (2016). Variation of Defensive Substance and Transcriptome Analysis of Different Resistant Pinus Massoniana Inoculated by pine Wood Nematode. Ph.D. Thesis.

[41] Funayama S., Sonoike K., Terashima I. (1997). Photosynthetic properties of leaves of Eupatorium makinoi infected by a geminivirus. Photosynth. Res..

[42] Robert G.N. (1986). The Biochemistry and Physiology of Plant Disease. BioScience.

[43] Yu L., Guo S., Zhu W., Yan J., Hei Y. (2011). Effects of tomato yellow leaf curl virus on photosynthetic characteristics and chloroplast ultra-structure of the tomato leaves. Acta Bot. Boreali-Occident. Sin..

[44] Skalamera D., Heath M.C. (1998). Changes in the cytoskeleton accompanying infection-induced nuclear movements and the hypersensitive response in plant cells invaded by rust fungi. Plant J..

[45] Clemente M., Corigliano M., Pariani S., Sánchez-López E., Sander V., Ramos-Duarte V. (2019). Plant Serine Protease Inhibitors: Biotechnology Application in Agriculture and Molecular Farming. Int. J. Mol. Sci..

[46] Navot N., Pichersky E., Zeidan M., Zamir D., Czosnek H. (1991). Tomato yellow leaf curl virus: A whitefly-transmitted geminivirus with a single genomic component. Virology.

[47] Hohlfeld K., Tomassi C., Wegner J.R.K., Kesteleyn B., Linclau B. (2015). Disubstituted BIS-THF moieties as new P2 ligands in nonpeptidal HIV-1 protease inhibitors. ACS Med. Chem. Lett..

[48] Li T., Huang Y., Xu Z.-S., Wang F., Xiong A.-S. (2019). Salicylic acid-induced differential resistance to the Tomato yellow leaf curl virus among resistant and susceptible tomato cultivars. BMC Plant Biol..

[49] Zhang H., Ma X.Y., Qian Y.J., Zhou X. (2010). Molecular characterization and infectivity of Papaya leaf curl China virus infecting tomato in China. J. Zhejiang Univ. Sci. B.

[50] Li Z.H., Zhou X.P., Zhang X., Xie Y. (2004). Molecular characterization of tomato-infecting begomoviruses in Yunnan, China. Arch. Virol..

[51] Lapidot M., Friedmann M., Pilowsky M., Ben-Joseph R., Cohen S. (2001). Effect of Host Plant Resistance to Tomato yellow leaf curl virus (TYLCV) on Virus Acquisition and Transmission by Its Whitefly Vector. Phytopathology.

[52] Hu L., Huang Z., Liu S., Fu J. (2012). Growth Response and Gene Expression in Antioxidant-related Enzymes in Two Bermudagrass Genotypes Differing in Salt Tolerance. J. Am. Soc. Hortic. Sci..

[53] Chahid K., Laglaoui A., Zantar S., Ennabili A. (2015). Antioxidant-enzyme reaction to the oxidative stress due to alpha-cypermethrin, chlorpyriphos, and pirimicarb in tomato (*Lycopersicon esculentum Mill.*). Environ. Sci. Pollut. Res. Int..

[54] Yang Y., Liu Q., Wang G.X., Wang X.D., Guo J.Y. (2010). Germination, osmotic adjustment, and antioxidant enzyme activities of gibberellin-pretreated Picea asperata seeds under water stress. New For..

[55] Yoshiyuki N., Kozi A. (1987). Purification of Ascorbate Peroxidase in Spinach Chloroplasts; Its Inactivation in Ascorbate-Depleted Medium and Reactivation by Monodehydroascorbate Radical. Plant Cell Physiol..

[56] Heath R.L., Packer L. (1968). Photoperoxidation in isolated chloroplasts. I. Kinetics and stoichiometry of fatty acid peroxidation. Arch. Biochem. Biophys..

[57] Gayoso C., Pomar F., Novo-Uzal E., Merino F., de Ilárduya O.M. (2010). The Ve-mediated resistance response of the tomato to Verticillium dahliae involves H_2_O_2_, peroxidase and lignins and drives PAL gene expression. BMC Plant Biol..

[58] Spagna G., Barbagallo R., Chisari M., Branca F. (2005). Characterization of a tomato polyphenol oxidase and its role in browning and lycopene content. J. Agric. Food Chem..

[59] Pastrana-Bonilla E., Akoh C.C., Sellappan S., Krewer G. (2003). Phenolic content and antioxidant capacity of muscadine grapes. J. Agric. Food Chem..

[60] Jia Z., Tang M., Wu J. (1999). The determination of flavonoid contents in mulberry and their scavenging effects on superoxide radicals. Food Chem..

[61] Love M.I., Huber W., Anders S. (2014). Moderated estimation of fold change and dispersion for RNA-seq data with DESeq2. Genome Biol..

[62] Chen S., Zhou Y., Chen Y., Gu J. (2018). fastp: An ultra-fast all-in-one FASTQ preprocessor. Bioinformatics.

[63] Langmead B., Salzberg S.L. (2012). Fast gapped-read alignment with Bowtie 2. Nat. Methods.

[64] Kim D., Langmead B., Salzberg S.L. (2015). HISAT: A fast spliced aligner with low memory requirements. Nat. Methods.

[65] Pertea M., Pertea G.M., Antonescu C.M., Chang T.C., Mendell J.T., Salzberg S.L. (2015). StringTie enables improved reconstruction of a transcriptome from RNA-seq reads. Nat. Biotechnol..

[66] Ashburner M., Ball C.A., Blake J.A., Botstein D., Butler H., Cherry J.M., Davis A.P., Dolinski K., Dwight S.S., Eppig J.T. (2000). Gene ontology: Tool for the unification of biology. The Gene Ontology Consortium. Nat. Genet..

[67] Kanehisa M., Goto S. (2000). KEGG: Kyoto encyclopedia of genes and genomes. Nucleic Acids Res..

[68] Subramanian A., Tamayo P., Mootha V.K., Mukherjee S., Ebert B.L., Gillette M.A., Paulovich A., Pomeroy S.L., Golub T.R., Lander E.S. (2005). Gene set enrichment analysis: A knowledge-based approach for interpreting genome-wide expression profiles. Proc. Natl. Acad. Sci. USA.

[69] Jing J., Yin-lei W., Li-ping Z., Rong Z., Ya-ru L., Tong-min Z., Wen-gui Y. (2017). Selection of tomato reference genes for qRT-PCR. Jiangsu J. Agric. Sci..

[70] Livak K.J.A., Schmittgen T.D.B. (2001). Analysis of Relative Gene Expression Data Using Real-Time Quantitative PCR and the 2 ^ΔΔ C T^ Method. Methods.

