# Supplementary figures and images for: Exogenous Nitric Oxide Alleviates the Damage Caused by Tomato Yellow Leaf Curl Virus in Tomato through Regulation of Peptidase Inhibitor Genes
Source: Int J Mol Sci. 2022 Oct 19;23(20):12542. doi: 10.3390/ijms232012542 (PMC9604136; doi:10.3390/ijms232012542)

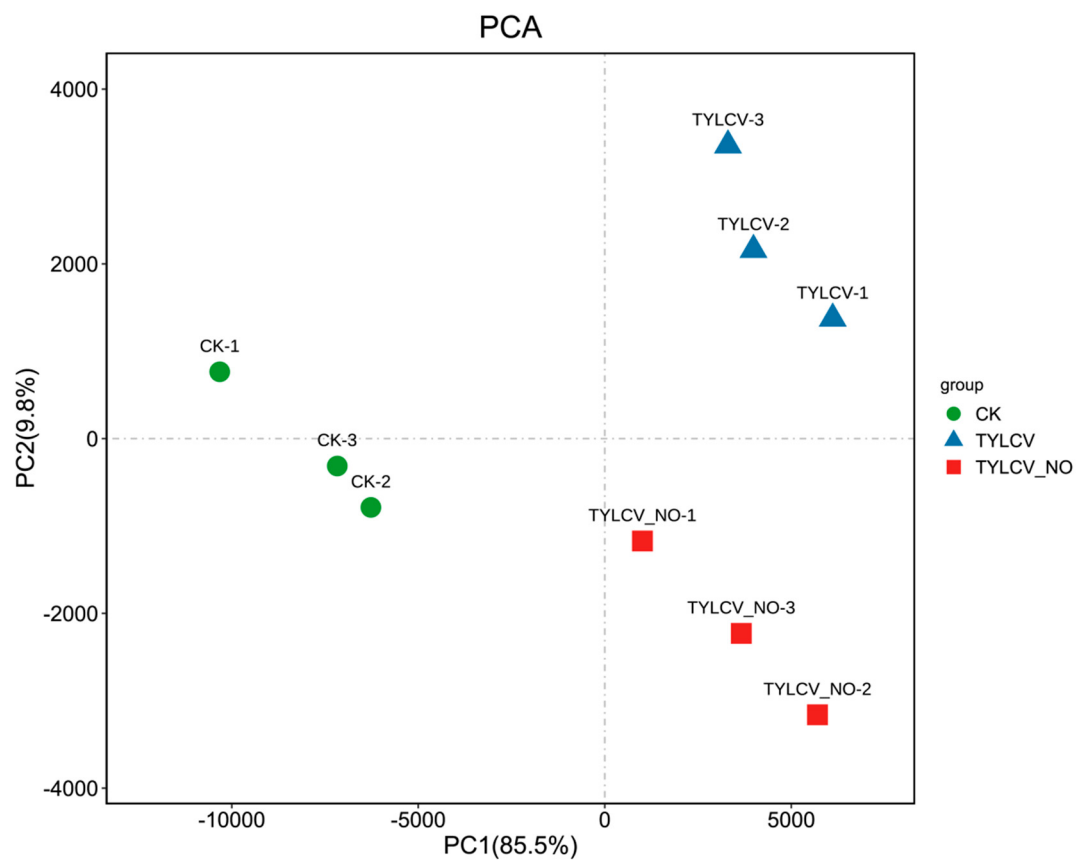

Figure S1 Sample principal component analysis

Supplement: Supplementary file 1 [file ijms-23-12542-s001.zip › Figure S1.pdf]
